# “Down syndrome: an insight of the disease”

**DOI:** 10.1186/s12929-015-0138-y

**Published:** 2015-06-11

**Authors:** Ambreen Asim, Ashok Kumar, Srinivasan Muthuswamy, Shalu Jain, Sarita Agarwal

**Affiliations:** Department of Medical Genetics, Sanjay Gandhi Post Graduate Institute of Medical Sciences, Lucknow, 226014 India

## Abstract

Down syndrome (DS) is one of the commonest disorders with huge medical and social cost. DS is associated with number of phenotypes including congenital heart defects, leukemia, Alzeihmer’s disease, Hirschsprung disease etc. DS individuals are affected by these phenotypes to a variable extent thus understanding the cause of this variation is a key challenge. In the present review article, we emphasize an overview of DS, DS-associated phenotypes diagnosis and management of the disease. The genes or miRNA involved in Down syndrome associated Alzheimer’s disease, congenital heart defects (AVSD), leukemia including AMKL and ALL, hypertension and Hirschprung disease are discussed in this article. Moreover, we have also reviewed various prenatal diagnostic method from karyotyping to rapid molecular methods -  MLPA, FISH, QF-PCR, PSQ, NGS and noninvasive prenatal diagnosis in detail.

## Introduction

Down syndrome is one of the most leading causes of intellectual disability and millions of these patients face various health issues including learning and memory, congenital heart diseases(CHD), Alzheimer’s diseases (AD), leukemia, cancers and Hirschprung disease(HD). The incidence of trisomy is influenced by maternal age and differs in population (between 1 in 319 and 1 in 1000 live births) [1-5]. DS has high genetic complexity and phenotype variability [6-8]. Trisomic fetuses are at elevated risk of miscarriages and DS people have increased incidence of developing several medical conditions [9]. Recent advancement in medical treatment with social support has increased the life expectancy for DS population. In developed countries, the average life span for DS population is 55 years [10].

**Figure 1:**
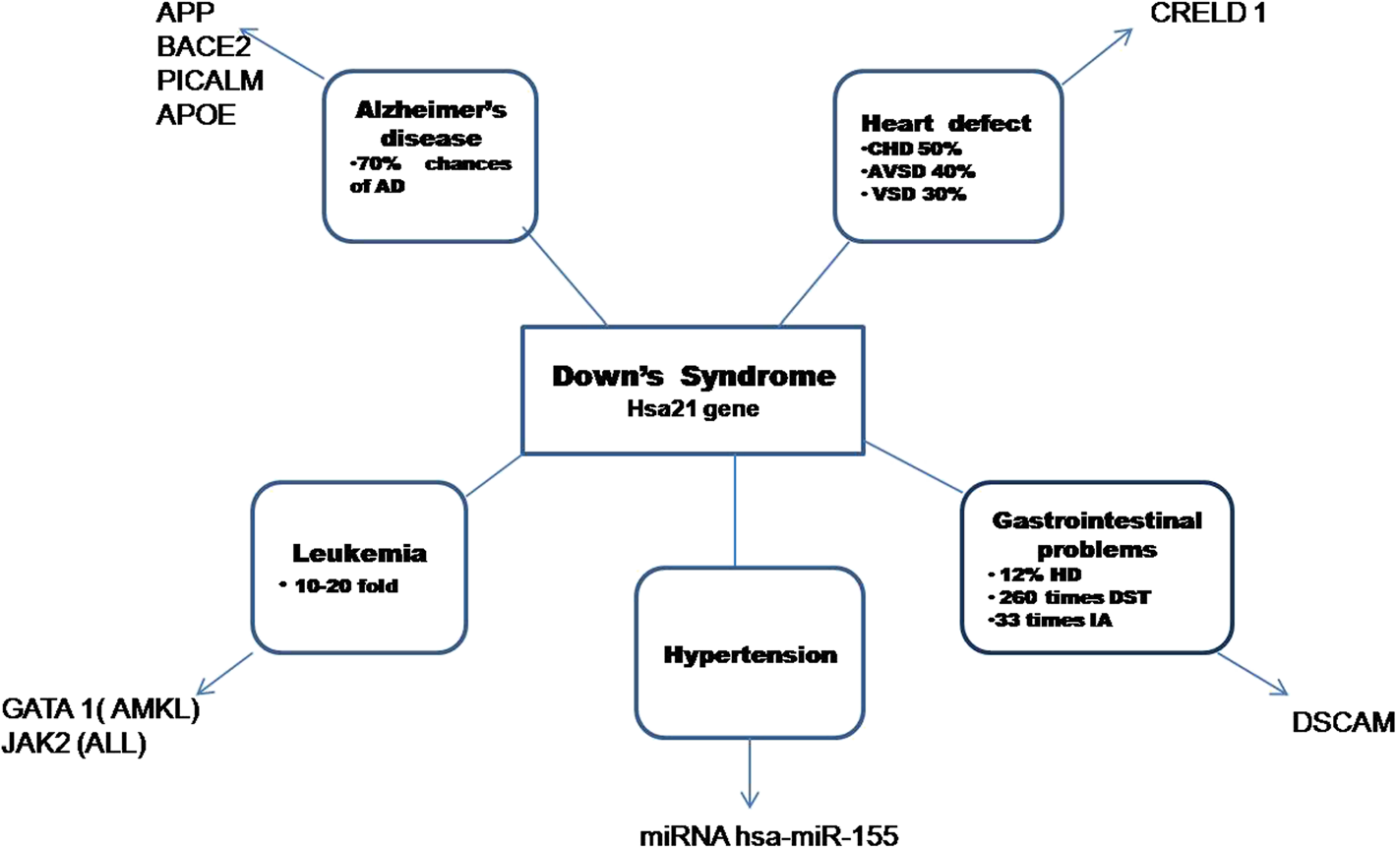
Various conditions associated with Downs’s syndrome with its causative genes.

## Review

### Human Chromosome 21

DS complex phenotype results from dosage imbalance of genes located on human chromosome 21(Hsa 21). The genetic nature of DS together with the relatively small size of Hsa 21 encouraged scientist to concentrate efforts towards the complete characterization of this chromosome in the past few years. The length of 21q is 33.5 Mb [11] and 21 p is 5–15 Mb [12]. A total 225 genes was estimated when initial sequence of 21q was published [11]. Hsa 21 has 40.06% repeat content out of which the repeat content of SINE’s, LINE’s, and LTR are 10.84%, 15.15%, 9.21% respectively. The Table 1 given below highlights some of the genes present on chromosome 21.

**Table 1:** **Some common gene present in chromosome 21**

**S. no.**	**Genes**	**Abbreviation**
1.	APP	Amyloid beta (A4) precursor protein
2.	C21 or f59:	Chromosome 21 open reading frame 59
3.	CBS	Cystathionine-beta-synthase
4.	CLDN14	Claudin 14
5.	HLCS	Holocarboxylase synthetase (biotin-(proprionyl-coenzyme a-carboxylase (ATP-hydrolysing)) ligase)
6.	KCNE1	Potassium voltage-gated channel, isk-related family, member 1
7.	KCNE 2	Potassium voltage-gated channel, isk-related family, member 2
8.	LAD	Leukocyte adhesion deficiency
9.	SOD 1	Superoxide dismutase 1
10.	TMPRSS3	Transmembrane protease, serine 3
11.	PCNT	Centrosomal pericentrin
12.	DSCR1	Down Syndrome critical region 1
13.	DYRK1A	Dual specificity tyrosine-(Y)-phosphorylation regulated kinase 1A
14.	RRPB1	Ribosomal RNA processing 1 homolog B
15.	S100B	Calcium binding protein

### Features of DS

There are various conserved features occurring in all DS population, including learning disabilities, craniofacial abnormality and hypotonia in early infancy [13]. Some people of DS are affected by variant phenotypes including atrioventricular septal defects (AVSD) in heart, leukemia’s (both acute megakaryoblastic leukemia(AMKL) and acute lymphoblastic leukemia(ALL)), AD and HD. DS individual have variety of physical characteristics like a small chin, slanted eye, poor muscle tone, a flat nasal bridge, a single crease of the palm and a protuding due to small mouth and large tongue [14]. Other features includes big toe, abnormal pattern of fingerprint and short fingers.

### Genetics of the disease

The most common cause of having a DS babies is presence extra copy chromosome 21 resulting in trisomy. The other causes can be Robertsonian translocation and isochromosomal or ring chromosome. Ischromosome is a term used to describe a condition in which two long arms of chromosome separate together rather than the long and short arm separating together during egg sperm development. Trisomy 21 (karyotype 47, XX, + 21 for females and 47, XY, + 21 for males) is caused by a failure of the chromosome 21 to separate during egg or sperm development. In Robertsonian translocation which occurs only in 2-4% of the cases, the long arm of the chromosome 21 is attached to another chromosome (generally chromosome 14). While mosaicism deals with the error or misdivision occurs after fertilization at some point during cell division. Due to this people with mosaic DS have two cell lineages which contribute to tissues and organs of individuals with Mosacism (one with the normal number of chromosomes, and other one with an extra number 21) [15].

### Genotype-phenotype correlation

Gene dosage imbalance hypothesis states that DS patients have an increased dosage or copy number of genes on Hsa 21 that may lead to an increase in gene expression [13-15]. This hypothesis has been extended to include the possibility that specific genes or subsets of genes may control specific DS phenotypes [16]. Amplified developmental instability hypothesis states that a non-specific dosage of a number of trisomic genes leads to a genetic imbalance that causes a great impact on the expression and regulation of many genes throughout the genome [13, 14]. Another hypothesis known as critical region hypothesis was also added to this list. Phenotypic analyses was done on individuals with partial trisomy for Hsa21 identified that only one or a few small chromosomal regions, termed “Down syndrome critical regions” (DSCR) a region of 3.8-6.5 Mb on 21q21.22, with approximately 30 genes responsible for the majority of DS phenotypes [15,16]. Previously a region of 1.6 to 2.5 Mb was recognised as sufficient cause for DS pehnotype [17, 18]. The sequencing of Hsa 21 proved to be an important factor in the progression of DS research [19] and led to further insight into genotype-phenotype correlations associated with DS and precise characterizations of DSCR regions [13]. A “critical region” within 21q22 was believed to be responsible for several DS phenotypes including craniofacial abnormalities, congenital heart defects of the endocardial cushions, clinodactyly of the fifth finger and mental retardation [20].

Dual-specificity tyrosine phosphorylation-regulated kinase (DYRK1A) and regulator of calcineurin 1 (RCAN1), Down syndrome cell adhesion molecule (DSCAM) has been suggested to play a critical role in the developing brain and has also been identified as a candidate gene for the increased risk of CHD in DS individuals [21,22]. DSCAM is a critical factor in neural differentiation, axon guidance, and the establishment of neural networks and it has been suggested that the disruption of these processes contributes to the DS neurocognitive phenotype [22]. Based on thorough analyses of studies on humans and DS mouse models, it is evident that there is not a single critical region of genes sufficient to cause all DS phenotypes. Alternatively, it is likely that there are multiple critical regions or critical genes contributing to a respective phenotype or group of phenotypes associated with DS [23].

### Various clinical conditions associated to Down syndrome

The various clinical conditions associated with DS are Alzheimer’s disease, heart defects, leukemia, hypertension and gastrointestinal problems (Figure 1). The molecular pathogenesis mechanism of these DS related phenotype must be studied along with its causative agents in order to have a better understanding of the disease. Below are some DS related phenotype discussed in detail which are as follows:

### Neurological problems

DS patients have greatly increased risk of early onset AD. After the age of 50, the risk of developing dementia increases in DS patients up to 70% [23-27]. There are various genes reported to cause early onset AD. Some of the genes described in the current literature are APP (amyloid precursor protein), BACE2 (beta secretase 2), PICALM (Phosphatidylinositol binding clathrin assembly protein) and APOE(Apolipoprotein E) etc. APP is an integral membrane protein which is concentrated in synapse of neurons and trisomy of this protein is likely to make significant contribution to the increased frequency of dementia in DS individuals. The triplication of Hsa 21 along with APP in people without DS has been recently shown to be associated with early onset AD. A tetranucleotide repeat, ATTT , in intron 7 of the amyloid precursor protein has been associated with the age of onset of AD in DS in a preliminary study [28]. Various mouse models are used to observe degeneration of basal forebrain cholinergic neurons (BFCNs). Ts65Dn mice is dependent on trisomy of APP expression of retrograde axonal transport [29]. Studies have also revealed that BACE2 which encodes enzyme beta secretase 2 is also involved in AD. APP and BACE 2 genes are located on chromosome 21. The current data on DS support the association of haplotypes in BACE2 with AD [30]. Besides APP and BACE2 genes, other genes like PICALM and APOE are also found to be associated with the age of onset of Alzheimer’s dimentia in DS [31].

### Cardiac problems

The incidence of CHD in newborn babies with DS is up to 50% [32]. Endocardial cushion defect also called as atrioventricular cushion defect is most common form which affects up to 40% of the patients. Ventricular septal defect (VSD) is also present in these population which affects up to 35% of the patients [33]. The essential morphological hallmark of an AVSD is the presence of a common atrioventricular junction as compared to the separate right and left atrioventricular junction in the normal heart. Other morphological features include defects of the muscular and membranous atrioventricular septum and an ovoid shape of the common atrioventricular junction. There is disproportion of outlet and inlet dimensions of the left ventricle, with the former greater than the latter as compared to the normal heart where both dimensions are similar [34]. While in case of VSD, the defect lies in ventricular septum of the heart due to which some of the blood from the left ventricle leaks into the right ventric leading to pulmonary hypertension. Mutation in non Hsa 21 CRELD1 (Cysteine rich EGF like domain1) gene contributes to the development of AVSD in DS [35]. CRELD1 is located on chromosome 3p25. It encodes a cell surface protein that functions as cell adhesion molecule and is expressed during cardiac cushion development. CRELD1 gene contains 11 exons spanning approximately 12 kb [36]. To the present, two specific genetic loci for AVSD have been identified. One was AVSD 1 locus present on chromosome 1p31-p21 [37]. Other locus was present on chromosome 3p25 and the corresponding gene was CRELD1 [36,38]. Maslen *et al*. in [33] have identified two heterozygous missense mutation (p.R329C and p.E414K) with two subjects in DS and AVSD. They have recruited 39 individual of DS with complete AVSD and have found the same mutations. In the same study, DNA of 30 individual of trisomy without CHD was studied for both mutations, no such mutation was identified [35]. R329C which was originally reported in an individual with sporadic partial AVSD and now it is also detected in individual of DS with AVSD. Interestingly, with the same mutation (p.R329C), the severity of heart defect was greater in patients of DS with AVSD. Thus, identification of CRELD 1 mutation in 2/39 individual (5.1%) of DS with complete AVSD suggests the defects in CRELD 1 contribute to pathogenesis of AVSD in context with trisomy 21.

### Hematological problems

Patients with DS display a unique spectrum of malignancies, which include leukemia’s as well as solid tumors. The first report of leukemia in a DS patient occurred in 1930 [39] and the first systematic study in 1957 [40]. Studies indicate that patients with DS have a 10–20 fold increased relative risk of leukemia, with a cumulative risk of 2% by age 5 and 2.7% by age 30 [41]. They constitute approximately 2% of all pediatric acute lymphoblastic leukemia(ALL) and approximately 10% of pediatric acute myeloid leukemia (AML). Leukemogenesis of acute megakaryoblastic leukemia (AMKL) in DS patients is associated with the presence of somatic mutations involving GATA 1 gene (or also called as GATA-binding factor 1) [42]. GATA 1 is a chromosome X- linked transcription factor which is essential for erythoid and megakaryocytic differentiation. Because of these GATA 1 mutations, there is a production of shorter GATA 1 protein thereby leading to uncontrolled proliferation of immature megakaryocytes [42,43]. On the other hand, acquired gain of function mutation in Janus Kinase 2 gene are present in approximately 30% of cases with ALL in DS [44,45].

### Hypertension

People with DS have been reported to have a reduced incidence of hypertension [46,47]. Trisomy of the Hsa21 microRNA hsa-miR-155 contributes to this [48]. Hsa-miR-155 is proposed to specifically target one allele of the type-1 angiotensin II receptor (AGTR1) gene, resulting in it’s under- expression, which contribute to a reduced risk of hypertension. Further studies are required to validate this hypothesis and determine whether other genes may also protect people with DS against hypertension.

### Gastrointestinal problems

DS patients constitute ~12% of all cases of HD. Duodenal stenosis (DST) and imperforate anus (IA) are 260 and 33 times more likely to occur DS [23,49]. HD is a form of low intestinal obstruction caused by the absence of normal myenteric ganglion cells in a segment of the colon [50]. In HD children, the absence of ganglion cells results in the failure of the distal intestine to relax normally. Peristaltic waves do not pass through the aganglionic segment and there is no normal defecation, leading to functional obstruction. Abdominal distention, failure to pass meconium, enterocolitis and bilious vomiting are the predominant signs and symptoms and appear within a few days after birth. Infants with duodenal atresia or DST present with bilious vomiting early in the neonatal period. If left untreated, it will result in severe dehydration and electrolyte imbalance. IA is a birth defects in which the rectum is malformed and it is associated with an increased incidence of some other specific anomalies as well, together being called the VACTERL association: vertebral anomalies, anal atresia, cardiovascular anomalies, tracheoesophageal fistula, esophageal atresia, renal and limb defects.

Alterations of approximately 10 non Hsa21 genes have been linked to this disease [51]. Several researches have shown that HD contain the DSCAM gene which is expressed in neural crest that give rise to enteric nervous system [49]. Overlapping critical region was described both for DST and IA [51]. No other Hsa21 genes have been implicated so far.

### Diagnostic methods

Prevention of DS depends upon offering prenatal diagnosis to high risk pregnancies via amniocentesis and chorionic villus sampling (CVS). Amniocentesis and CVS are quite reliable but offers risk of miscarriage of between 0.5 to 1% [52]. Based soft markers like small or no nasal bone, large ventricles and nuchal fold thickness, the risk of DS for fetus can be identified through ultrasound generally at 14 to 24 weeks of gestation [53]. Increased fetal nuchal translucency indicates an increased risk of DS [54]. The other methods used for prenatal diagnosis in which traditional cytogenic analysis is still widely used in different countries. However some rapid molecular assays-FISH(fluorescent in situ hybridization), QF-PCR (quantitative fluorescence PCR), and MLPA(multiplex probe ligation assay)- also used for prenatal diagnosis.

### Routine karyotyping

Cytogenetic analysis of metaphase karyotype remains the standard practice to identify not only trisomy 21, but also all other aneuploidies and balanced translocations. Details on diagnostic methods with advantages and disadvantages are mentioned in Table 2.

**Table 2:** **Common techniques used for diagnosis of Down’s syndrome along with its advantages and disadvantages**

	**Method**	**Description**	**Advantages**	**Disadvantages**
1	**Cytogenetics analysis**	Giemsa banding (G-banding) is performed on fetal cells at metaphase stage on amniocytes (grown in vitro) or CVS.	• Suitable for low income countries where physician can be presumed to have acquired a high level of diagnostic skill in the absence of laboratory services.	• Time consuming.
				• Resolution of special importance for the detection of structural. abnormalities may be quite low as the spontaneous dividing cells are more condensed than those obtained after cell culture in vitro.
				• In CVS, occurrence of confined placental mosaicism and occurrence of aberrant cells that do not represent the status of fetus.
				• Chances of giving a false positive and false negative result.
2	**FISH(Fluorescence in situ hybridization)**	FISH involves hybridization of selected chromosome specific DNA sequences that have been labeled with fluorescent dye to chromosome preparation. The fluorescently labeled sequences stick to corresponding DNA of chromosome and can be visualized under microscope.	• As it uses smaller probes thus the signals appears to be more distinct as dots.	• Sometimes diffused signals are obtained because it uses chromosome at interphase stage which appears less condense than those of metaphase.
			• It uses higher number of interphase nuclei for analysis, so the problem of any suspected mosaicism is resolved.	• Time consuming since it involves preparation of slides, fluorescent microscopy and spot counting (~30min per sample is expected).
				• Maternal and fetal XX is not distinguished by FISH.
3	**QF-PCR (Quantitative fluorescent-polymerase chain reaction)**	Involves amplification and detection of STR using fluorescently labeled primers. The product is thus visualized and quantified as peaks areas of respective length using an automated DNA sequencer with Gene Scan software.	• Highly reliable and reproducible.	• Poses a challenge in the case of mosaicism.
			• Chances of getting false negative and false positive cases are rare.	• While testing sex chromosome abnormalities samples from normal XX female may show homozygous QF-PCR pattern indistinguishable from those produced by sample with single X as in Turner syndrome.
			• Maternal contamination is easily detected.	
			• Faster approach as it can give the diagnosis within 24 hours.	
4	**Paralogous sequence quantification (PSQ)**	A PCR based method for detection of targeted chromosome number abnormalities, based on the use of paralogous genes. Paralogous sequences have high degree of sequence identity but accumulate nucleotide substitution in a locus specific manner. These differences are called as paralogous sequence mismatches which can be quantified using pyrosequencing.	• The first generation design of test requires 10 separate PCR reaction per sample, which significantly reduces the sample throughput and increases the probability of handling errors.	• Expensive when compared to others.
			• It can handle 30–40 samples in a day and report result in less than 48 hours.	
5.	**MLPA (multiplex probe ligation assay)**	MLPA is based on hybridization and PCR method. Divided into 4 phases: DNA denaturation, hybridization of probe to the complementary target sequence, probe ligation and PCR amplification of ligated probe. These amplified products are analysed through capillary electropheresis.	• Very short time for diagnosis (2–4 days).	• Unable to exclude low level placental and true mosaicism.
			• Relatively low costs	
6.	**NGS (Next Generation Sequencing)**	Clonally amplified DNA templates are sequenced in a massively parallel. It provides a digital quantitative information, in that each sequence read is a countable “sequence tag” representing an individual clonal DNA template or a single DNA molecule.	• The current time for sample processing, sequencing, and data interpretation in experienced hands is 5 to 8 days.	• The cost of sequencing is approximately $700 –$1000 per sample.
				• Complex data analysis.

### Rapid aneuploidy testing methods

Over the past 10 years however, several other methods have been developed and used for the rapid detection of trisomy 21, either in fetal life or after birth. The most widely used is FISH of interphase nuclei, using Hsa 21-specific probes or whole-Hsa 21 [55]. An alternative method that is now widely used in some countries is QF-PCR, in which DNA polymorphic markers (microsatellites) on Hsa 21 are used to determine the presence of three different alleles [56]. This method relies on informative markers and the availability of DNA. Rapid diagnosis by PCR-based methods using polymorphic STR markers may reduce these difficulties using conventional approach. Using STR markers method we can detect trisomy in 86.67% cases with only two markers. Using more number of markers can further increase the reliability of the test. Simultaneously parental origin of the nondysjunction can also be detected [57,58]. Additional method to measure copy number of DNA sequences include MLPA [59] which was first introduced in 2002 as a method of relative quantification in DNA. MLPA offers various advantages like – a very short time for diagnosis (2–4 days), effectiveness, simplicity and relatively low costs. It is based on hybridization and PCR method and is divided into four steps: DNA denaturation, hybridization of probe to the complementary target sequence, probe ligation and PCR amplification. And finally capillary electrophoresis of PCR amplified products is carried out. However MLPA is unable to exclude low level placental and true mosaicism [60].

### Advancement in the diagnosis

A recent method, termed paralogous sequence quantification (PSQ), uses paralogous sequences to quantify the Hsa 21 copy number. PSQ is a PCR based method for the detection of targeted chromosome number abnormalities termed paralogous sequence quantification (PSQ), based on the use of paralogous genes. Paralogous sequences have a high degree of sequence identity, but accumulate nucleotide substitutions in a locus specific manner. These sequence differences, which are termed as paralogous sequence mismatches (PSMs), can be quantified using pyrosequencing technology, to estimate the relative dosage between different chromosomes. PSQ is a robust, easy to interpret, and easy to set up method for the diagnosis of common aneuploidies, and can be performed in less than 48 h, representing a competitive alternative for widespread use in diagnostic laboratories. The sequencing is quantitatively done by using pyrosequencing [61]. Finally, comparative genomic hybridization (CGH) on BAC chips can be used for the diagnosis of full trisomy or monosomy, and for partial (segmental) aneuploidies [62,63].

### Noninvasive Prenatal diagnosis

Fetal cells in maternal ciruculation: Ever since the discovery of presence of fetal lymphocytes in maternal blood was made in 1969, the investigators are trying to develop genetics-based noninvasive prenatal diagnostics (NIPD) [64]. Despite several advantages offered by this approach, the use of fetal cells for NIPD has never reached clinical implementation because of their paucity (on the order of a few cells per milliliter of maternal blood) and concerns of fetal cell persistence in the maternal circulation between pregnancies.

Cell free fetal DNA from maternal serum: This novel approach was proposed in 1997. Cell-free fetal DNA constitutes between 5% and 10% of the total DNA in maternal plasma and increases during gestation and rapidly clears from the circulation post delivery. Several clinical applications based on the analysis of cell-free fetal DNA have been developed like determining fetal Rh D status in Rh D-negative women [65], sex in sex-linked disorders [66,67], and detection of paternally inherited autosomal recessive and dominant mutations [68]. However, there remains the outstanding challenge of the use of cell-free fetal DNA for the detection of chromosomal aneuploidy, in particular trisomies 21, 18, and 13. Several approaches have been adopted like the origin of circulating cell-free fetal DNA is primarily the placenta, whereas maternal cell-free DNA is derived from maternal leukocytes [69]. The approach includes studying differences in genomic DNA methylation between the placenta and paired maternal leukocytes, investigators have characterized placenta-specific epigenetic markers [70] and also finding of circulating cell-free placenta-derived mRNA allowed the identification of placenta-specific mRNA production [71].

The concept of digital PCR was also introduced to serve the same purpose. In digital PCR, individual fetal and maternal circulating cell-free DNA fragments are amplified under limiting-dilution conditions and the total number of chromosome 21 amplifications (representing maternal plus fetal contributions) divided by the number of reference chromosome amplifications should yield a ratio indicating an over- or underrepresentation of chromosome 21.

Although the digital PCR approach is conceptually solid, the low percentage of cell-free fetal DNA in the maternal plasma sample requires the performance of thousands of PCRs to generate a ratio with statistical confidence. This can be overcome by using of multiple target amplifications and enrichment of cell-free fetal DNA which are still under research trail.

Next recent method added to the list is next generation sequencing (NGS) which is based on the principle of clonally amplified DNA templates (or, most recently, single DNA molecules) are sequenced in a massively parallel fashion within a flow cell [72,73]. NGS provides digital quantitative information, in which each sequence read is a countable “sequence tag” representing an individual clonal DNA template or a single DNA molecule. This quantification allows NGS to expand the digital PCR concept of counting cell-free DNA molecules.

Fan et al. and Chiu et al. in 2008 described noninvasive detection of trisomy 21 by NGS [74]. Both groups extracted cell-free DNA from maternal plasma samples from both euploid and trisomy pregnancies. DNA from each sample was sequenced on the Illumina Genome Analyzer, and each sequence read was aligned to the reference human genome. Chiu et al. build on their earlier work with the Illumina Genome Analyzer and demonstrate noninvasive NGS-based trisomy 21 detection with the sequencing-by-ligation approach on the Life Technologies SOLiD platform [75]. Cell-free DNA was extracted from 15 pregnant women, 5 of whom carried trisomy 21 fetuses and it was clonally amplified by emulsion PCR, and sequenced in 1 chamber of an 8-chamber SOLiD slide. This process yielded a median of 59 × 10^6^ 50-base reads per sample. A median of 12 × 10^6^ reads (or 21%) were each aligned uniquely to one location of the reference human genome (with masking of repeat regions), for a coverage of approximately 20% of the haploid human genome. For each trisomy 21 case, the chromosome 21 z score value indicated a 99% chance of a statistically significant difference from the chromosome 21 z scores for the controls. As reported earlier with the Illumina Genome Analyzer, a nonuniform distribution of aligned sequence reads was observed with the SOLiD data.

The current time for sample processing, sequencing, and data interpretation in experienced hands is 5 to 8 days for the Genome Analyzer and SOLiD platforms respectively with the cost of approximately $700 – $1000 per sample. Going forward, one can expect streamlining and automation of technical processes and data analysis, coupled with reduced sequencing costs.

Ultimately, reduced sequencing costs and turnaround times could pave the way for NGS-based NIPD to be considered as an alternative to serum biomarker screening, which,while cost-effective remains prone to false positives. Forty years after the discovery of circulating fetal cells, the vision of NIPD appears clearer and closer.

### Management of the disease

One of the hallmarks of DS is the variability in the way that the condition affects people with DS. With the third 21st chromosome existing in every cell, it is not surprising to find that every system in the body is affected in some way. However, not every child with DS has the same problems or associated conditions. Parents of children with DS should be aware of these possible conditions so they can be diagnosed and treated quickly and appropriately. The goal of the study is to point out the most common problems of which parents should be aware.

Timely surgical treatment of cardiac defects during first 6 months of life may prevent from serious complications. Congenital cataracts occur in about 3% of children and must be extracted soon after birth to allow light to reach the retina. A balance diet and regular exercise are needed to maintain appropriate weight. Feeding problems and failure to thrive usually improve after cardiac surgery. A DS child should have regular check up from various consultants. These include:

- Clinical geneticist - Referral to a genetics counseling program is highly desirable
- Developmental pediatrician
- Cardiologist - Early cardiologic evaluation is crucial for diagnosing and treating congenital heart defects, which occur in as many as 60% of these patients
- Pediatric pneumonologist -Recurrent respiratory tract infections are common in patients with DS
- Ophthalmologist
- Neurologist/Neurosurgeon – As many as 10% of patients with DS have epilepsy; therefore, neurologic evaluation may be needed
- Orthopedic specialist
- Child psychiatrist - A child psychiatrist should lead liaison interventions, family therapies, and psychometric evaluations
- Physical and occupational therapist
- Speech-language pathologist
- Audiologist

## Conclusion

DS or Trisomy 21, being the most common chromosomal abnormality among live born infants, is associated with a number of congenital malformations. Several theories have been put forward to increase our understanding in phenotype and genotype correlation. A “critical region” within 21q22 was believed to be responsible for several DS phenotypes including craniofacial abnormalities, congenital heart defects of the endocardial cushions, clinodactyly of the fifth finger and mental retardation and several other features. The primary goal of this review is to unravel the common genes involved in DS associated phenotypes, including APP, BACE2, PICALM, APOE, GATA 1, JAK 2, CRELD 1 and DSCAM. This reviews also provides the detailed description on the application of techniques to prenatal diagnosis in DS. Rapid aneuploidy testing has been introduced in mid 1990’s in the form of FISH where testing can be done on uncultured amniocytes. Within a couple of years, MLPA and QF-PCR has been added in the list of rapid aneuploidy testing. The other methods includes: NGS for cell free fetal DNA screening for maternal plasma. Except ,FISH, MLPA and QF-PCR other method are not commercialized for aneuploidy diagnosis due to their running cost, labor intensive protocol and complex data analysis. Since various clinical conditions are associated with DS, hence the management of these patients requires an organized multidisciplinary approach and continuous monitoring of these patients which has been discussed in this review article.
